# Editorial: Secondary metabolites and metabolism in tea plants

**DOI:** 10.3389/fpls.2023.1143022

**Published:** 2023-02-14

**Authors:** Zhaoliang Zhang, Chuankui Song, Jian Zhao, Enhua Xia, Weiwei Wen, Lanting Zeng, Vagner A. Benedito

**Affiliations:** ^1^ State Key Laboratory of Tea Plant Biology and Utilization, School of Tea and Food Science and Technology, Anhui Agriculture University, Anhui, China; ^2^ Key Laboratory of Tea Science of Ministry of Education, College of Horticulture, Hunan Agricultural University, Changsha, China; ^3^ Key Laboratory of Horticultural Plant Biology (MOE), College of Horticulture and Forestry Sciences, Huazhong Agricultural University, Wuhan, China; ^4^ South China Botanical Garden, Chinese Academy of Sciences (CAS), Guangzhou, China; ^5^ School of Agriculture and Food, Davis College of Agriculture, Natural Resources and Design, West Virginia University, Morgantown, WV, United States

**Keywords:** *Camellia sinensis*, specialized metabolism of plants, tea quality, plant biochemistry, natural products

Tea is the world’s most popular prepared beverage. It is prepared from the delicate shoots of the tea plant (*Camellia sinensis*, Theaceae). It is one of the most important cash crops in the world, with 60% of tea produced by smallholders in low-income nations (FAO, 2022). The sustainability of tea production is currently challenged by climate change and the excessive use of chemical fertilizers, herbicides, and pesticides. Tea plants synthesize secondary metabolites to survive under various stresses such as extreme temperature, UV radiation, drought, nutrient deficiency, pathogen infection, and herbivore feeding. Moreover, the complex flavor and aroma and the health-promoting properties of tea are provided by the large amounts and abundant types of secondary metabolites in tea leaves, which are the reason for the popularity of teas. Catechins, theanine, caffeine, volatiles, and their derivatives are the most critical and prominent secondary metabolites for tea quality.

Secondary metabolites accumulate due to dynamic biosynthesis, storage, transport, and decomposition. Secondary metabolism, as in other plant species, is developmentally and environmentally controlled, but there is still much to learn about its regulatory mechanisms in tea. Knowing more about them will allow us to use them to manage secondary metabolism in tea and develop better cultivation tactics for improved beverage quality. This special issue is an important contribution to the field by reporting on substantial advances in understanding the molecular basis of secondary metabolism in tea plants. This special issue has 16 papers, 15 of which are original research reports on various aspects of tea metabolite storage, transport, signal transduction, and metabolic control.

After the tea leaves are picked, they undergo a series of processes to become the final product. Post-harvest processing varies depending on the variety of tea produced. For example, black tea and oolong tea are made from leaves that have been withered or partially dried before the oxidation step. During oxidation, enzymes in the leaves react with oxygen in the air, changing the color and flavor of the leaves. The leaves are then dried to halt the oxidation process. Indeed, oolong tea gets its characteristic floral aroma from fatty acid-derived volatiles formed during post-harvest processing. Zhou et al. investigated changes in alcohol dehydrogenase activity, gene expression, and the fatty acid metabolome in oolong tea leaves during processing. The data showed that oxygen supply during processing coordinates the metabolic flow of C6 aldehydes of the LOX-HPL pathway and that an oxidative micro-environment leads to enzymatic reactions forming distinctive acids and derivative esters of this tea type.

Phenylpropanoids encompass a broad family of secondary metabolites found in plants, whose primary precursor is phenylalanine and, to a lesser extent, tyrosine. This class consists of five chemical groups (flavonoids, lignin, phenolic acids, stilbenes, and coumarins), some of which have antioxidant and anti-inflammatory features that benefit human health. Tea contains a high concentration of phenylpropanoids, including galloylated catechins and the bioactive flavonoid epigallocatechin gallate. On the other hand, the biosynthesis of galloylated catechins is poorly known. Li et al. investigated 118 R2R3-MYB transcription factor genes in the tea genome. They discovered nine genes whose expression levels were enriched in buds and growing leaves, which contain the most galloylated catechins. Three of these genes were discovered to be connected to key biosynthetic genes for epigallocatechin gallate and galloylated catechins, making them excellent candidates for further functional studies.

Aside from their health benefits, flavonoids contribute significantly to the astringent tea flavor. Flavonols are flavonoids with a unique B-ring structure (3-hydroxyflavone). Shi et al. investigated the metabolic features of phenolic chemicals throughout flower development using metabolomic and transcriptome studies. Their studies demonstrated that the flavonol content of flowers increased with development, with petals and stamens being the primary locations of flavone and flavonol accumulation. Moreover, the expression of *CsFLSb* was considerably different in fertile and sterile flowers. This suggests that flavonols influence fertility. The study also identified three *CsFLS* genes with diverse roles in vegetative and reproductive organs. This research underscores the importance of balancing vegetative and reproductive growth in tea plants.

Purple tea has a distinctive color, flavor, and enhanced health value due to high levels of flavonoids and anthocyanins. Song et al. discovered that a reduction in flavonoids and anthocyanin concentrations caused leaves of a purple variety to turn green. Although the mechanisms of flavonoid and anthocyanin synthesis have been studied in several purple tea cultivars, the molecular regulation of these activities during leaf growth and color variation remains unclear. The authors discovered regulatory genes and networks involved in flavonoid production in purple tea plants using an integrated transcriptome and metabolome study, indicating that the purple color is due to the co-pigmentation of quercetin and kaempferol derivatives. They also discovered many genes that could be involved in flavonoid and anthocyanin accumulation in purple leaves. This study adds to our understanding of the metabolic and molecular mechanisms behind the formation of flavonoids and anthocyanins in tea plants.

Gallic acid is a polyphenolic molecule present in tea with antioxidant and anti-inflammatory effects. According to Zhang et al., spraying gallic acid on tea plants activated defense mechanisms against caterpillars by stimulating jasmonic acid signaling and the synthesis of various phenylpropanoid compounds. These results suggest that gallic acid is an effective inducer of direct defense responses in tea plants against caterpillars. This study will help us better understand the relationship between plants and herbivore insects and aid in developing cultivation protection strategies.

Color plays a critical role in tea quality. Shading is a common technique used in the cultivation of tea plants to improve the quality of the tea leaves. Shading is typically achieved using netting or other materials that block some sunlight from reaching the tea plants. Increased chlorophyll content *via* shading improves tea leaf color *via* unknown mechanisms. Additionally, Chen et al. explored the effect of shading on the expression of genes involved in chlorophyll synthesis in tea plants. They found that decreased light intensity induces gene expression of a protochlorophyllide oxidoreductase (CsPOR), leading to increased chlorophyll content. The study also identified three transcription factors as possible regulators of this process and some key interactions. The content of other metabolites is also affected by shading. Shao et al. employed non-targeted metabolomics to examine the metabolic pathways in the shoots and roots of two tea cultivars under shade and relighting conditions over the summer. The amino acid synthase genes *CsGS1.1* and *CsTSI* were induced in the leaves and roots of both cultivars. These findings reveal the effects of shading on carbon and nitrogen metabolism in both leaves and roots and that root metabolism influences leaf metabolism to increase tea quality.

Fang et al. investigated how phytohormone and transcriptome profiles of tea leaves changed when black, blue, and red net shading was used. They discovered that blue net shading enhanced bud density, fresh weight of 100 buds, and yield much more than black net shading while having an equivalent effect on flavonoid content. The different shading treatments affected phenylpropanoid biosynthesis, MAPK signaling, and hormone signal transduction, as revealed by the transcriptome profiles. The levels of salicylic acid and melatonin correlated with light signal perception and signaling genes, and several genes had stronger interactions with phytohormone biosynthetic genes. These results suggest that different shading affects the development and physiology of tea plants through the regulation of phytohormone levels.

Zhou et al. found five calmodulin-binding transcription activator (CAMTA) genes in the tea genome and categorized them into three classes. They were detected in several organs under various stress treatments. Co-expressed genes were identified in cold and dry conditions, forming five distinct co-expression networks centered on CAMTA genes. Cold treatment boosted hormone regulation, transcriptional control, and protein processing-related pathways, whereas drought increased hormone, lipid, and carbon metabolism. Their protein interaction network study showed that CAMTAs bind to distinct cis-elements in target gene promoters and control their transcription. These findings pave the way for further studies into the involvement of CAMTA genes in stress responses in tea plants.

Jiang et al. used an untargeted metabolomics method to investigate relationships between genes and metabolites in fresh shoots of 68 tea accessions. Their findings highlight the intricate interactions between non-volatile metabolite content and gene expression levels. This work will contribute to a better understanding of the genetic control of metabolites in fresh tea shoots.

Geraniol is a volatile monoterpene alcohol that contributes to the aroma of tea. According to Zhou et al., it is present in tea leaves as geranyl-primeveroside. Geranyl-primeveroside levels vary in tea leaves depending on age and tissue color, with younger, greener leaves having higher levels than older, darker leaves. The study also identified and described two geraniol synthase genes and two Nudix hydrolase genes in the tea genome and found that CsNUDX1-cyto is more efficient at converting geranyl-pyrophosphate to geranyl-monophosphate *in vitro* than CsNUDX1-chlo.

According to Shan et al., the older the tea plant, the better the scent and taste of the tea it produces, becoming more nuanced and sweeter with less astringency. They reported that gene expression and metabolite levels differed among the leaves of younger and older plants. These findings imply that secondary metabolic pathways, such as flavonoid biosynthesis, may play a role in the variations in tea quality across plants of different ages.

Non-proteogenic amino acids play important roles in plant development and stress response. Theanine is a prominent glutamine analog in green tea that may have antidepressant and soothing qualities. It influences tea quality and price by adding an umami flavor to the beverage. Its physiological role is not well understood. Feng et al. identified and characterized CsCAT2, a theanine transporter localized to the tonoplast that is involved in the transport and storage of theanine in the roots throughout the winter when theanine is stored. Its expression decreases in the spring during theanine transfer from roots to shoots. Chen et al. reported that salt stress increased the levels of theanine and other amino acids in new shoots. This increase was associated with the upregulation of genes involved in theanine biosynthesis. When applied to new shoots, theanine increased tolerance to salt stress in tea plants and Arabidopsis and increased antioxidant activity in shoots under salt stress. These findings suggest that theanine plays a role in salt stress tolerance in tea plants *via* a redox homeostasis pathway.

E3 ubiquitin ligases were studied in the tea genome by Xing et al.. They discovered 335 genes encoding RING finger proteins, with 53 responding to stress. When one of them (*CsMIEL1*) was overexpressed in Arabidopsis, it decreased tolerance to methyl jasmonate and reduced anthocyanin accumulation in response to cold.

Finally, Chen reviewed the role of the leaf cuticle in tea quality. Given its role in preventing water loss in the plant, as opposed to the withering procedure used to reduce water loss after processing, the cuticle’s influence on tea quality is undefined. This review delves into numerous elements of cuticle structure and composition, as well as knowledge gaps and open questions that could be future study topics.

The seven topic editors of this special issue would like to express their heartfelt gratitude to the 125 authors and many peer reviewers who contributed to this special issue. We are indebted to the many peer reviewers who generously provided their expertise and time to read and provide feedback on the manuscripts. We are confident that this volume will serve to advance tea research by shedding light on the mechanisms contributing to the production of the rich aroma and complex secondary metabolite profile of this popular beverage consumed daily by billions of people worldwide.

## Author contributions

All authors listed have made a substantial, direct, and intellectual contribution to the work and approved it for publication.
